# Dose-Volume Histogram Parameters and Quality of Life in Patients with Prostate Cancer Treated with Surgery and High-Dose Volumetric-Intensity-Modulated Arc Therapy to the Prostate Bed

**DOI:** 10.3390/cancers15133454

**Published:** 2023-06-30

**Authors:** Luca Hanke, Hongjian Tang, Christina Schröder, Paul Windisch, Ken Kudura, Mohamed Shelan, André Buchali, Stephan Bodis, Robert Förster, Daniel R. Zwahlen

**Affiliations:** 1 Department of Radiation Oncology, Cantonal Hospital Winterthur, Brauerstrasse 15, 8401 Winterthur, Switzerland; 2 Department of Nuclear Medicine, Sankt Clara Hospital, Kleinriehenstrasse 30, 4058 Basel, Switzerland; 3 Department of Radiation Oncology, Inselspital, University Hospital Bern, Freiburgstrasse 18, 3010 Bern, Switzerland; 4 Department of Radiation Oncology, University Hospital Ruppin-Brandenburg, Fehrbelliner Strasse 38, 16816 Neuruppin, Germany; 5 Department of Radiation Oncology, Cantonal Hospital Aarau, Tellstrasse 25, 5001 Aarau, Switzerland

**Keywords:** radiotherapy, quality of life, prostate cancer, prostate bed, dose-volume histogram, dose constraints

## Abstract

**Simple Summary:**

We looked at the long-term quality of life of patients treated with modern radiotherapy for relapsed prostate cancer after initial surgery. By comparing the original radiotherapy treatment plans with the reported quality of life of the patients, we looked for correlations between the doses received by the healthy pelvic organs and patients’ long-term quality of life. In contemporary radiotherapy practice, healthy organs are spared as much as possible from the high doses required to treat the cancer in order to avoid side effects. We were able to show that patient’s quality of life may also be impacted by low doses to their pelvic organs. Therefore, it seems desirable that specific thresholds need to be established in the low dose spectrum as well in order to further improve patients’ quality of life after radiotherapy. Since this is a complex endeavor requiring extensive research, we provide future researchers with our entire dataset in order to hopefully facilitate their work.

**Abstract:**

Introduction: Prostate bed radiotherapy (RT) is a major affecter of patients’ long-term quality of life (QoL). To ensure the best possible outcome of these patients, dose constraints are key for optimal RT planning and delivery. However, establishing refined dose constraints requires access to patient-level data. Therefore, we aimed to provide such data on the relationship between OAR and gastrointestinal (GI) as well as genitourinary (GU) QoL outcomes of a homogenous patient cohort who received dose-intensified post-operative RT to the prostate bed. Furthermore, we aimed to conduct an exploratory analysis of the resulting data. Methods: Patients who were treated with prostate bed RT between 2010 and 2020 were inquired about their QoL based on the Expanded Prostate Cancer Index Composite (EPIC). Those (n = 99) who received volumetric arc therapy (VMAT) of at least 70 Gy to the prostate bed were included. Dose-volume histogram (DVH) parameters were gathered and correlated with the EPIC scores. Results: The median age at the time of prostate bed RT was 68.9 years, and patients were inquired about their QoL in the median 2.3 years after RT. The median pre-RT prostate-specific antigen (PSA) serum level was 0.35 ng/mL. The median duration between surgery and RT was 1.5 years. The median prescribed dose to the prostate bed was 72 Gy. A total of 61.6% received prostate bed RT only. For the bladder, the highest level of statistical correlation (*p* < 0.01) was seen for V10-20Gy, Dmean and Dmedian with urinary QoL. For bladder wall, the highest level of statistically significant correlation (*p* < 0.01) was seen for V5-25Gy, Dmean and Dmedian with urinary QoL. Penile bulb V70Gy was statistically significantly correlated with sexual QoL (*p* < 0.05). A larger rectal volume was significantly correlated with improved bowel QoL (*p* < 0.05). Sigmoid and urethral DVH parameters as well as the surgical approach were not statistically significantly correlated with QoL. Conclusion: Specific dose constraints for bladder volumes receiving low doses seem desirable for the further optimization of prostate bed RT. This may be particularly relevant in the context of the aspiration of establishing focal RT of prostate cancer and its local recurrences. Our comprehensive dataset may aid future researchers in achieving these goals.

## 1. Introduction

Developing dose constraints for organs at risk (OARs) to reduce toxicities has been a key focus in radiation oncology research, from early consensus papers to the Quantitative Analyses of Normal Tissue Effects in the Clinic (QUANTEC) publications [1,2]. In the years post-QUANTEC, a variety of systematic reviews have tried to provide even more detailed recommendations. However, these efforts are limited by the lack of accessible patient-level data, which makes comparisons between different publications and meta-analyses difficult [3].

Optimizing post-operative radiotherapy (RT) to the prostate bed has been a hot topic in recent years because improving outcome and toxicity are both of great relevance for patients’ long-term quality of life (QoL). In this setting, various highly regarded publications have investigated timing, prescription dose, extent of treatment volume and added value of prostate-specific membrane antigen (PSMA) positron emission tomography/computed tomography (PET/CT), as well as necessity and choice of androgen deprivation therapy (ADT) [4,5,6,7,8,9,10,11]. The introduction of intensity-modulated radiotherapy (IMRT) and volumetric arc therapy (VMAT) has led to a more widespread adaptation of dose-intensified RT of the prostate bed in Switzerland and other countries without high-level evidence for improved outcome and held isotoxicity. At our institutions, we also followed this approach prior to the publication of the results of the SAKK 09/10 randomized phase 3 trial [8].

Our goal was, therefore, to provide patient-level data on the relationship between OAR doses and gastrointestinal (GI) as well as genitourinary (GU) QoL in patients who received dose-intensified post-operative RT (either adjuvant or salvage) to the prostate bed only. In addition, we present the results from an exploratory analysis of the resulting data.

## 2. Methods

**Figure 1:**
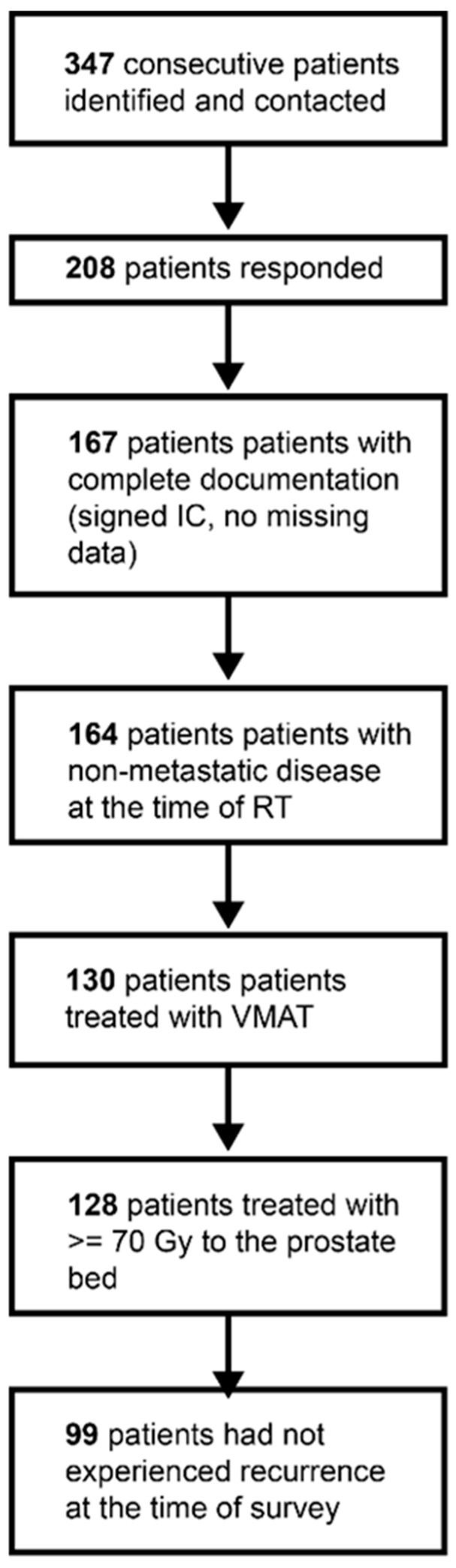
Inclusion workflow. Abbreviations: IC—informed consent, RT—radiation therapy, VMAT—volumetric intensity-modulated arc therapy.

The inclusion workflow is depicted in Figure 1. Three hundred and forty-seven consecutive patients who received RT for prostate cancer in the post-operative setting at our institution between 2010 and 2020 were identified and received the German versions of two QoL questionnaires via mail in August 2020, unless a date of death had been documented in our electronic health record. The questionnaires used were the Expanded Prostate Cancer Index Composite (EPIC) and the International Prostate Symptom Score (IPSS) [12,13,14]. The results of the former are presented herein. In addition, we asked the patients for information regarding the physician that they were currently seeing for the prostate cancer follow-up and sent related questions regarding the current disease status to them.

We received a response from 208 patients. From 167 of these patients, we obtained their QoL questionnaires, had treatment plans to export dose-volume histogram (DVH) parameters and obtained informed consent. Most contours (bladder, rectum, bowel, sigmoid, penile bulb and femoral heads) had been created during routine clinical practice. Additional contours (e.g., bladder wall, urethra, anterior and posterior rectum) were created by a medical student (L.H.) and were reviewed by a board-certified radiation oncologist (C.S. or R.F.) at the time of analysis.

To generate a homogeneous cohort in terms of RT parameters, only patients who had no signs of metastases at the time of RT (n = 164), had been treated with VMAT (n = 130), had been prescribed at least 70 Gy to the entire prostate bed (n = 128) and did not have signs of recurrence (neither clinical nor biochemical) at the last documented follow-up (n = 99) were included. Regarding the surgical technique, open, laparoscopic and robotic-assisted prostatectomy with or without nerve sparing were allowed and included for analysis.

Data preprocessing, analysis and visualization were performed via Python (version 3.9.7) using the numpy (version 1.20.3), pandas (version 1.3.4) and seaborn (0.11.2) packages.

Correlation coefficients were calculated using the Pearson correlation coefficient provided by the pandas.corr method. The *p*-value of the correlation was calculated using the scipy.stats.pearsonr method.

Institutional review board approval was obtained from the ethical review committee of the canton of Zurich for a project (project number: BASEC 2020-02112) to analyze the effects and side effects of radiotherapy at our institution (ClinicalTrials.gov Identifier: NCT05192876). Written informed consent for the analysis of anonymized clinical and imaging data was obtained from all patients, and all data were gathered in accordance with the World Medical Association Declaration of Helsinki: Research involving human subjects.

## 3. Results

The patient characteristics are provided in Table 1. The full patient-level dataset is provided in Supplementary Table S1. The median age at diagnosis was 64.9 years (range: 50.8–77.6 years), the median age at surgery was 65.2 years (range: 51.1–77.8 years), the median age at radiotherapy was 68.9 years (range: 51.6–83.4 years) and the median age at the time of the survey was 72.1 years (range: 53.4–85.6 years). The median duration between surgery and radiotherapy was 1.5 years (range: 0.1–17.7 years), and the median follow-up after radiotherapy (i.e., the time from radiotherapy to the survey) was 2.3 years (range: 0.2–7.6 years).

Fifty-five patients (55.5%) had previously undergone robotic-assisted, twenty-two patients (22.2%) had undergone open and sixteen patients (16.1%) had undergone laparoscopic prostatectomy. In six patients, the surgical technique was unknown. In 53 patients (53.5%), the surgery was classified as nerve-sparing, while in 36 patients (36.3%), it was not. In the remaining ten patients (10.1%), it was unclear whether nerve-sparing had been performed.

**Figure 2:**
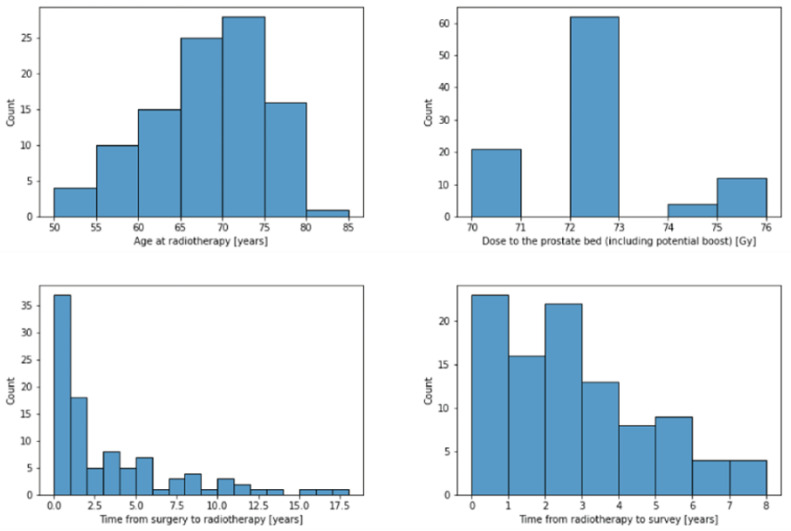
Histograms to illustrate the distributions of selected patient characteristics.

The median pre-RT prostate-specific antigen (PSA) serum level PSA was 0.35 ng/mL (range: 0–12.2 ng/mL). The patients were treated in fractions of either 1.8 Gy (n = 42, 42.4%) or 2 Gy (n = 57, 57.6%). The total dose to the prostate bed, including any boost volumes, ranged from 70 to 76 Gy with a median of 72 Gy. Thirty-eight patients (38.4%) received prostate bed and pelvic lymph node irradiation, while the remaining sixty-one patients (61.6%) received irradiation of the prostate bed alone. Histograms for selected patient characteristics are presented in Figure 2.

**Figure 3:**
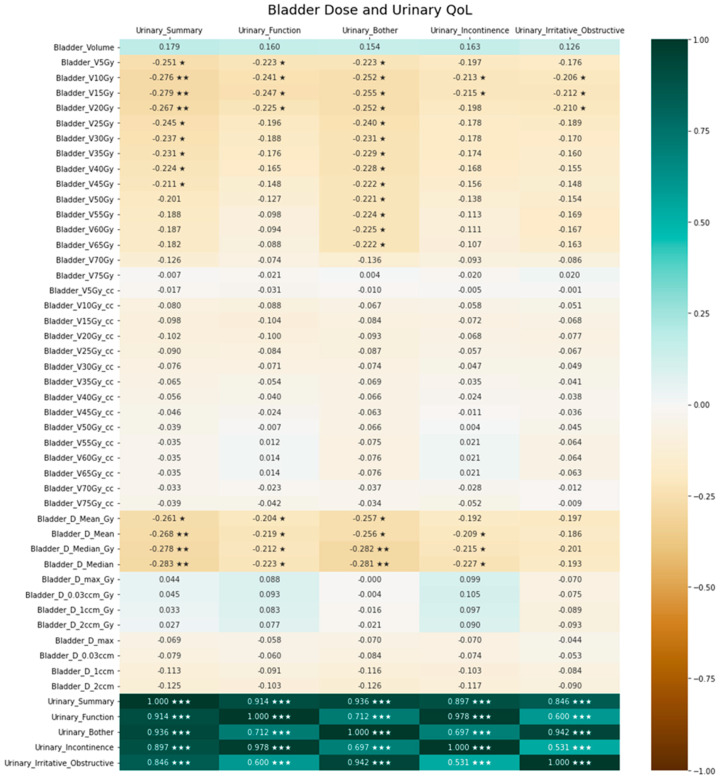
Association between bladder dose and urine-related quality of life. Positive correlation coefficients indicate a positive correlation, negative coefficients indicate a negative correlation and 0 indicates no correlation. The significance level of the correlation coefficients is indicated by asterisks (* denotes *p* < 0.05, ** denotes *p* < 0.01 and *** denotes *p* < 0.001).

**Figure 4:**
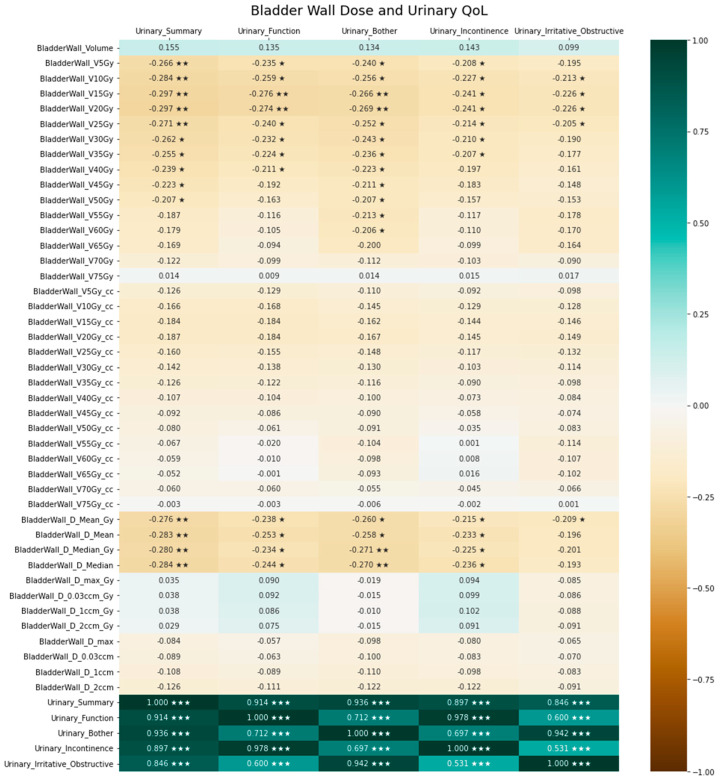
Association between bladder wall dose and urine-related quality of life. Positive correlation coefficients indicate a positive correlation, negative coefficients indicate a negative correlation and 0 indicates no correlation. The significance level of the correlation coefficients is indicated by asterisks (* denotes *p* < 0.05, ** denotes *p* < 0.01 and *** denotes *p* < 0.001).

**Figure 5:**
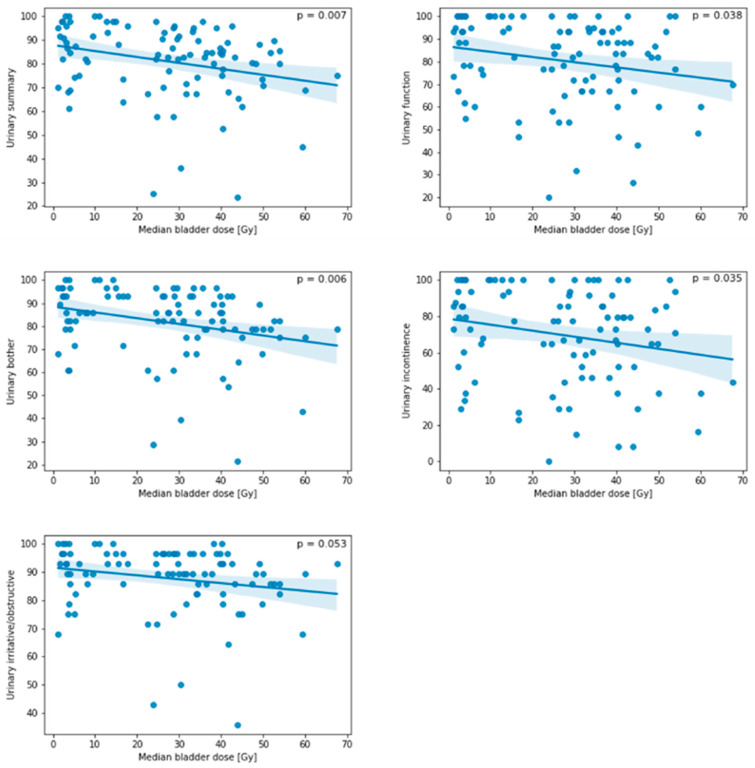
Scatterplots of the median bladder dose [Gy] and different quality of life domains (urinary summary, urinary function, urinary bother, urinary incontinence and urinary irritative/obstructive symptoms). The line indicates the fit of a linear regression model, while translucent bands indicate the 95% confidence interval. While there was a significant correlation between the median bladder dose with urinary summary, urinary function, urinary bother and urinary incontinence, the correlation between the median bladder dose and irritative/obstructive symptoms did not reach significance.

Exploratory analyses resulted in significant correlations of several DVH parameters regarding bladder as well as bladder wall dose with urinary QoL. This is illustrated by the bladder dose correlation heatmap in Figure 3, while the heatmap for the bladder wall is presented in Figure 4. Figure 5 provides exemplary scatterplots correlating the median bladder dose with the five urinary QoL domains (urinary summary, urinary function, urinary bother, urinary incontinence and urinary irritative/obstructive).

The urethral DVH parameters did not result in significant correlations with urinary QoL, and the corresponding heatmap is also presented in the Supplementary Material (Supplemental Figure S1). Increased penile bulb V70Gy was correlated with worse EPIC sexual bother (Supplemental Figure S2).

Correlation heatmaps for the rectal, anterior rectal wall, posterior rectal wall and sigmoid dose with gastrointestinal quality of life are provided in the Supplementary Material and show a significant correlation between a larger rectal volume and an improved QoL (Supplemental Figures S3–S6).

**Figure 6:**
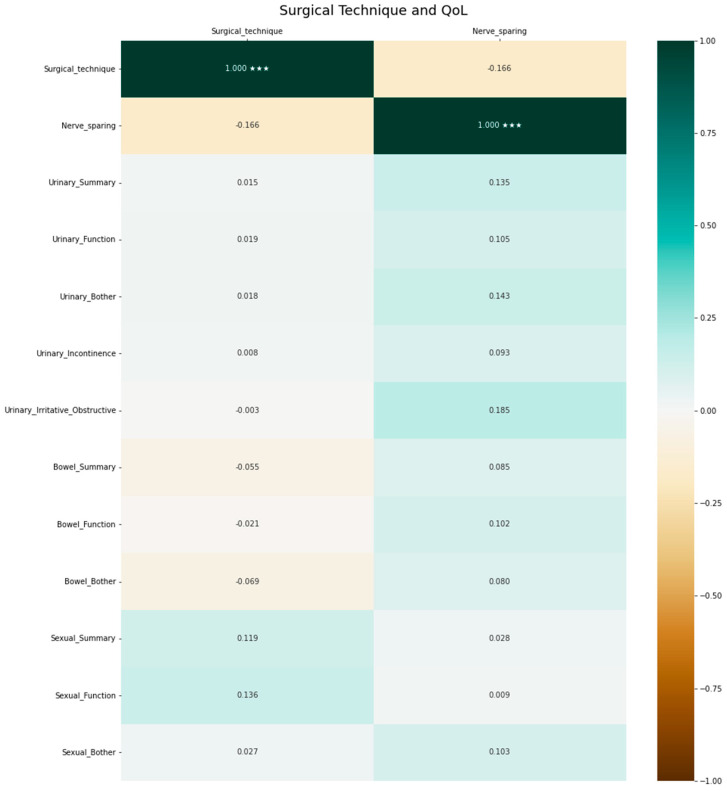
Association between surgical approach and quality of life. Positive correlation coefficients indicate a positive correlation, negative coefficients indicate a negative correlation and 0 indicates no correlation. The significance level of the correlation coefficients is indicated by asterisks (*** denotes *p* < 0.001).

The surgical approach (invasiveness and nerve-sparing) was not correlated with QoL. The respective heatmap is depicted in Figure 6.

## 4. Discussion

### 4.1. Summary of Study Objectives and Cohort

We aimed to identify the DVH parameters correlated with long-term QoL after prostatectomy and post-operative high-dose VMAT to the prostate bed. Patients had a median age of 68.9 years at the time of RT and RT was conducted in a median of 1.5 years after prostatectomy, therefore, typically in the salvage setting. About half of the patients had undergone robotic surgery, and nerve-sparing was also conducted in about half of the patients. As it was the national standard in Switzerland to deliver higher doses to the prostate bed before the publication of the SAKK 09/10 trial, all of the patients had received at least 70 Gy. According to institutional guidelines at the time, the recommended dose to the entire prostate bed was 72 Gy, and a further focal boost of any detectable local recurrence was strongly encouraged [8]. The prostate bed was contoured according to the European Organisation for Research and Treatment of Cancer (EORTC) guidelines, and post-operative RT was delivered as VMAT in all patients [15].

### 4.2. Bladder Volumes and QoL

In our analyses, bladder dose was significantly correlated with long-term EPIC-based QoL. As was to be expected, EPIC-based long-term QoL decreased with increasing median bladder dose in almost all urinary-specific domains. Interestingly, specific bladder and bladder wall DVH parameters were correlated with various urinary QoL domains. While this was to be expected for bladder and bladder wall Dmean and Dmedian, we detected that low-dose parameters, i.e., bladder V10Gy, bladder V15Gy, bladder V20Gy, bladder wall V5Gy, bladder wall V10Gy, bladder wall V15Gy, bladder wall V20Gy and bladder wall V25Gy, showed a significant negative correlation with the EPIC urinary summary. Traditionally, volumes receiving higher doses (V40Gy and beyond) have been associated with compromised QoL and increased late GU and GI toxicity [16,17,18,19]. Therefore, contemporary clinical practice requires the constraining of bladder volumes receiving higher doses. According to our knowledge, there are no recommended constraints for volumes receiving lower doses. In the SAKK 09/10 trial, it was required to keep the bladder wall V65Gy lower or equal to 50% [8]. In the RADICALS trial, V50Gy < 80% and V60Gy < 50% were used as bladder constraints [20]. The Veterans Affairs Radiation Oncology expert panel recommends bladder V65Gy < 50% and bladder V40Gy < 70% for conventional post-prostatectomy radiotherapy, and according to QUANTEC, bladder V65Gy < 50%, bladder V70Gy < 35%, bladder V75Gy < 25% and bladder V80Gy < 15% are recommended to circumvent increased late toxicity [21,22]. Our institution adhered to the QUANTEC constraints during plan optimization. Rigorous adherence to the QUANTEC planning objectives and the stringent use of VMAT with 10 MV may have circumvented increased late toxicity as intended, but also led to increased low doses received by the OARs [23]. In turn, this may have led to our finding that volumes receiving low doses are correlated with long-term urinary QoL. This could also explain why we did not find any stronger correlations between DVH parameters, particularly those for OAR volumes receiving high doses, except for penile bulb V70Gy, and any of the EPIC-based QoL items. However, this would then be true for most institutions utilizing current dose constraints and intensity-modulated techniques.

However, online adaptive radiotherapy yields great potential for improving the toxicity profile of pelvic RT because OAR doses can be substantially reduced [24].

### 4.3. Rectal Volumes and QoL

Our finding of larger rectal volumes being significantly correlated with improved QoL may simply be attributable to the fact that smaller OAR volumes of the OARs were irradiated when the OARs were larger.

### 4.4. Penile Bulb and QoL

Higher penile bulb doses have been correlated with erectile dysfunction in clinical trials of RT for prostate cancer [25,26]. Furthermore, D60–70% < 70Gy is one of the constraints for the penile bulb suggested by QUANTEC to ensure <55% severe erectile dysfunction. Therefore, our finding of an increased penile bulb V70Gy being correlated with a worse EPIC sexual bother score is not surprising, but consequently also illustrates that smaller CTV to PTV margins may have been justified in order to avoid an unnecessary dose to the penile bulb.

### 4.5. Relevance of the Study

In general, improving OAR sparing is not easily achievable in prostate-bed-only radiotherapy or pelvic radiotherapy, since it remains challenging to ensure the consistency of bladder and rectal filling despite standardized protocols for daily image-guided radiotherapy.

Overall, the findings of our work point toward the necessity for the establishment of dose constraints for bladder and bladder wall volumes receiving lower doses in addition to the existing high-dose constraints, which could also be investigated in current trials of dose de-escalation for prostate cancer. Our results may also be of particular relevance for current endeavors of establishing focal radiotherapy of prostate cancer (ClinicalTrials.gov identifier: NCT05616650) or prostate bed recurrences (ClinicalTrials.gov identifier: NCT05746806), as well as for the further optimization of re-irradiation after previous prostate cancer RT [27,28].

### 4.6. Strengths and Weaknesses

The strengths of this study include the homogenous, high-dose (>70 Gy) cohort and the use of a comprehensive, disease-specific QoL questionnaire. In addition, the inclusion of the entire dataset in Supplementary Table S1 enables the aggregation of our data as part of larger systematic reviews and meta-analyses in the future. The study is limited by its retrospective design and by the fact that the DVH parameters can only be correlated with indicators of QoL and not specific symptoms such as pain, bleeding, obstruction, erectile dysfunction, etc.

## 5. Conclusions

Specific dose constraints for bladder volumes receiving low doses seem desirable for the further optimization of prostate bed RT. This may be particularly relevant in the context of the aspiration of establishing focal RT of prostate cancer and its local recurrences. Our comprehensive dataset may aid future researchers in achieving these goals.

## Figures and Tables

**Table 1:** Patient characteristics. Abbreviations: IQR = interquartile range, PSA = prostate-specific antigen, RT = radiotherapy.

Characteristics	Mean	Median	Range	IQR
Age [years]				
At diagnosis	64.4	64.9	50.8–77.6	61.0–68.5
At surgery	64.8	65.2	51.1–77.8	61.4–69.3
At radiotherapy	68.3	68.9	51.6–83.4	64.6–72.9
At survey	71.0	72.1	53.4–85.6	66.6–75.9
Follow-up after radiotherapy [years]	2.7	2.3	0.2–7.6	1.2–4.0
Pre-RT PSA [ng/mL]	0.90	0.35	0.0–12.2	0.21–0.64
Total dose to the prostate bed [Gy]	71.8	72	70–76	72–72
Dose per fraction [Gy]	1.92	2	1.8–2	2–2

## Data Availability

The full dataset is provided in Supplementary Table S1.

## Supplementary Materials

The following supporting information can be downloaded at: https://www.mdpi.com/article/10.3390/cancers15133454/s1, Figure S1. Association between urethra dose and urinary quality of life; Figure S2. Association between penile bulb dose and sexual quality of life; Figure S3. Association between rectal dose and bowel-related quality of life; Figure S4. Association between anterior rectum dose and bowel-related quality of life; Figure S5. Association between posterior rectum dose and bowel-related quality of life; Figure S6. Association between sigmoid dose and bowel-related quality of life; Table S1: full patient-level dataset.
