# Supplementary material for: Dose-Volume Histogram Parameters and Quality of Life in Patients with Prostate Cancer Treated with Surgery and High-Dose Volumetric-Intensity-Modulated Arc Therapy to the Prostate Bed
Source: Cancers (Basel). 2023 Jun 30;15(13):3454. doi: 10.3390/cancers15133454 (PMC10340614; doi:10.3390/cancers15133454)
Supplement: Supplementary file 1 [file cancers-15-03454-s001.zip › Prostate_DVH_QoL_supplement_r1.pdf]

Supplemental Materials

In all supplemental figures, asterisks indicate the significance level of the correlation indices:

*	$p < 0.05$
**	$p < 0.01$
***	$p < 0.001$

Supplemental Figure S1. Association between urethra dose and urinary quality of life.

Supplemental Figure S2. Association between penile bulb dose and sexual quality of life.

Supplemental Figure S3. Association between rectal dose and bowel-related quality of life.

Supplemental Figure S4. Association between anterior rectum dose and bowel-related quality of life.

Supplemental Figure S5. Association between posterior rectum dose and bowel-related quality of life.

Supplemental Figure S6. Association between sigmoid dose and bowel-related quality of life.

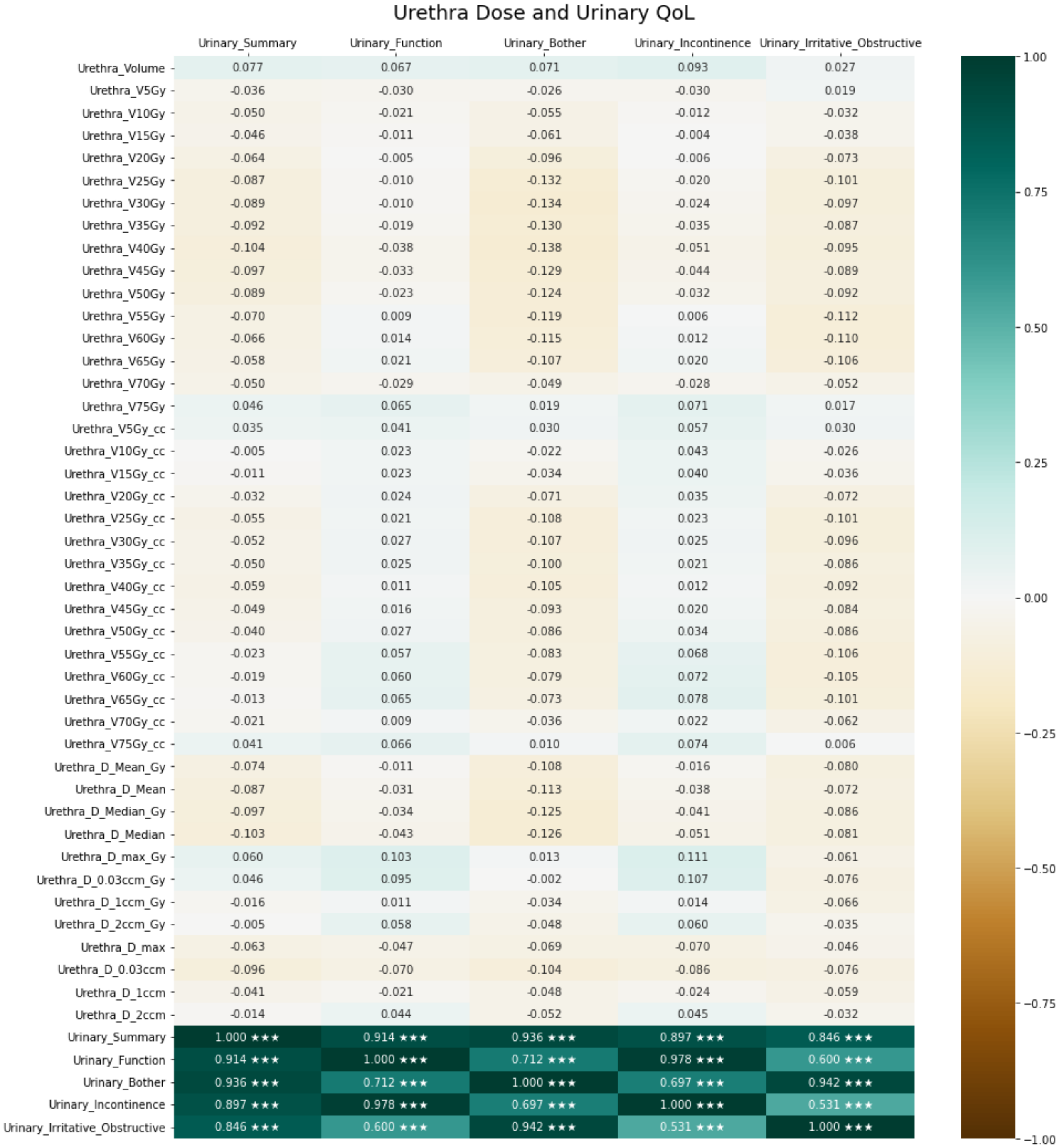

Supplemental Figure S1. Association between urethra dose and urinary quality of life.

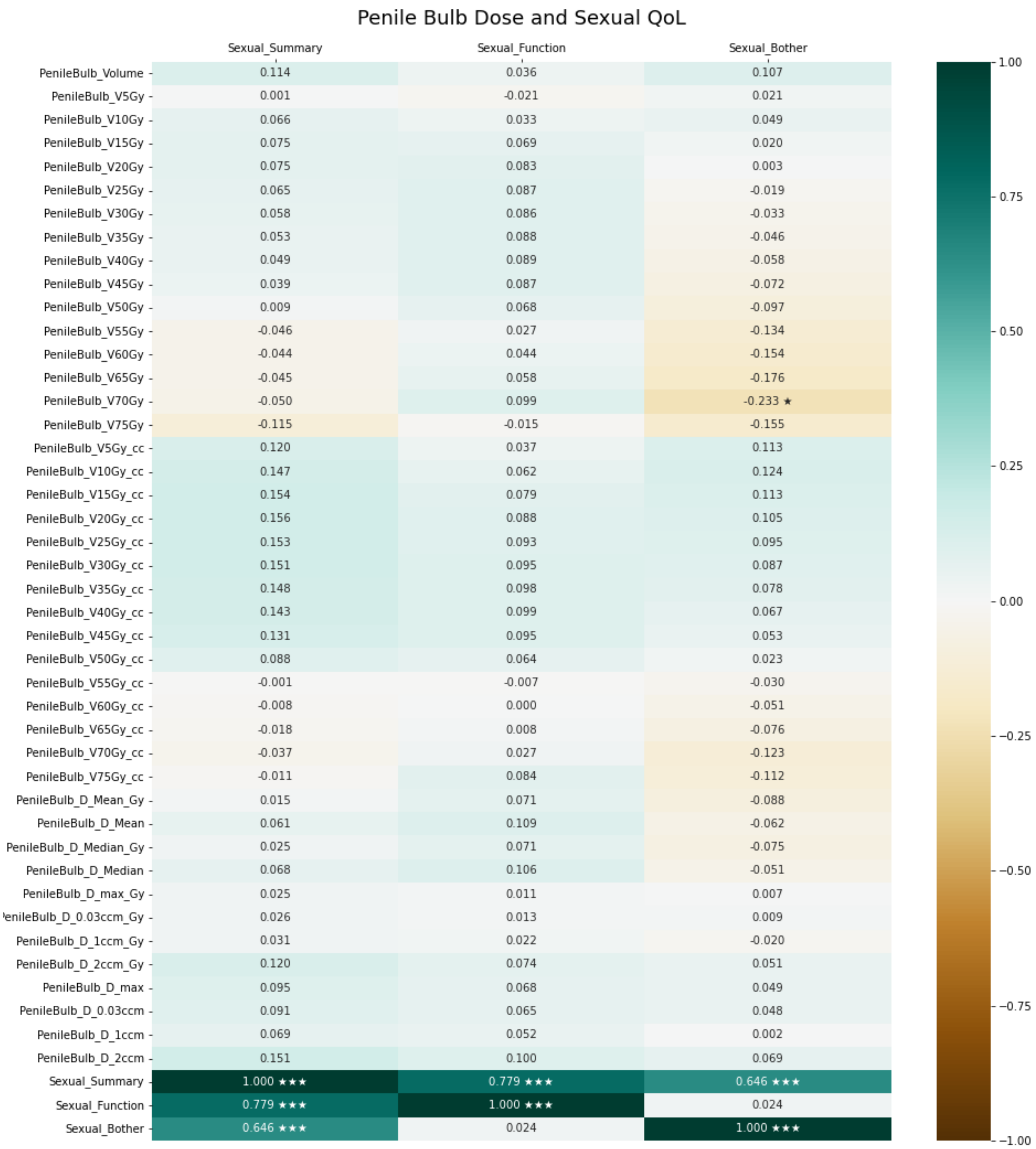

Supplemental Figure S2. Association between penile bulb dose and sexual quality of life.

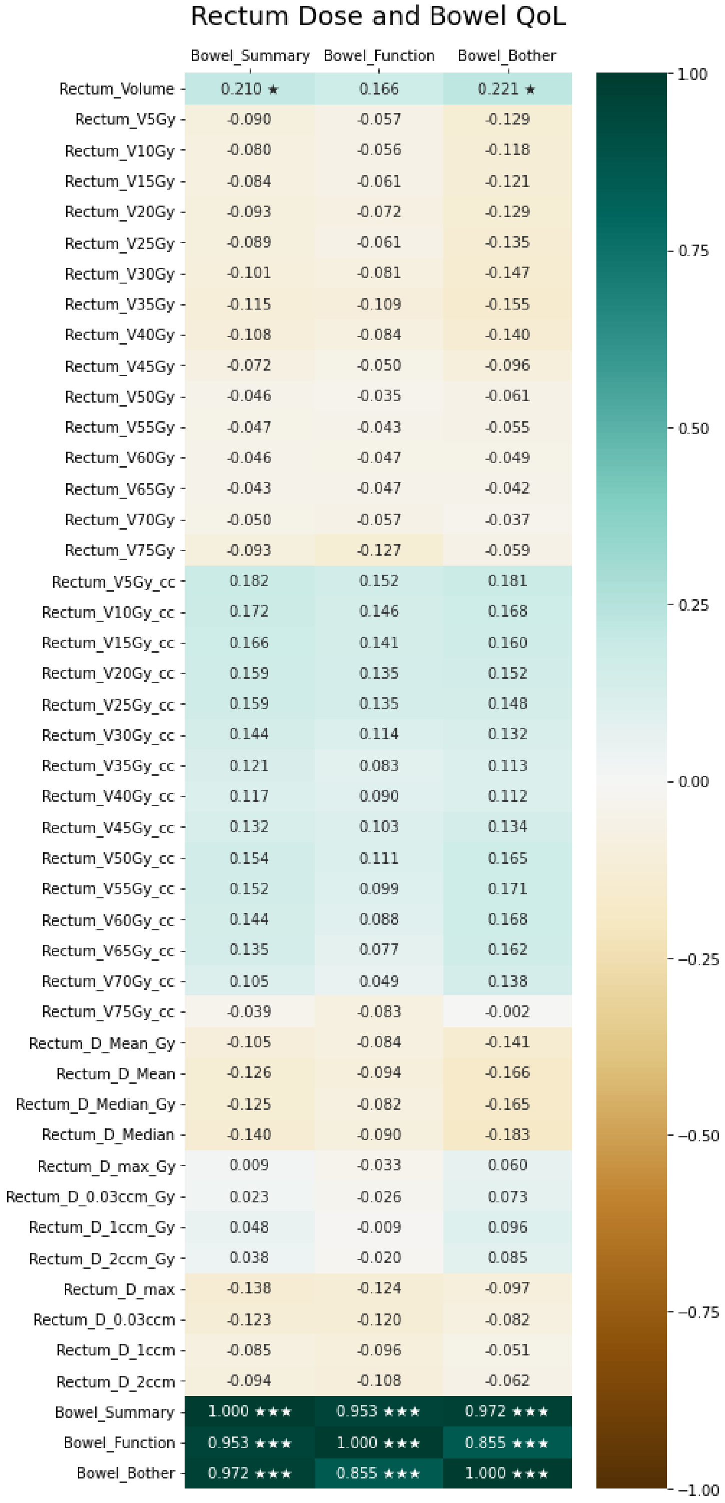

Supplemental Figure S3. Association between rectal dose and bowel-related quality of life.

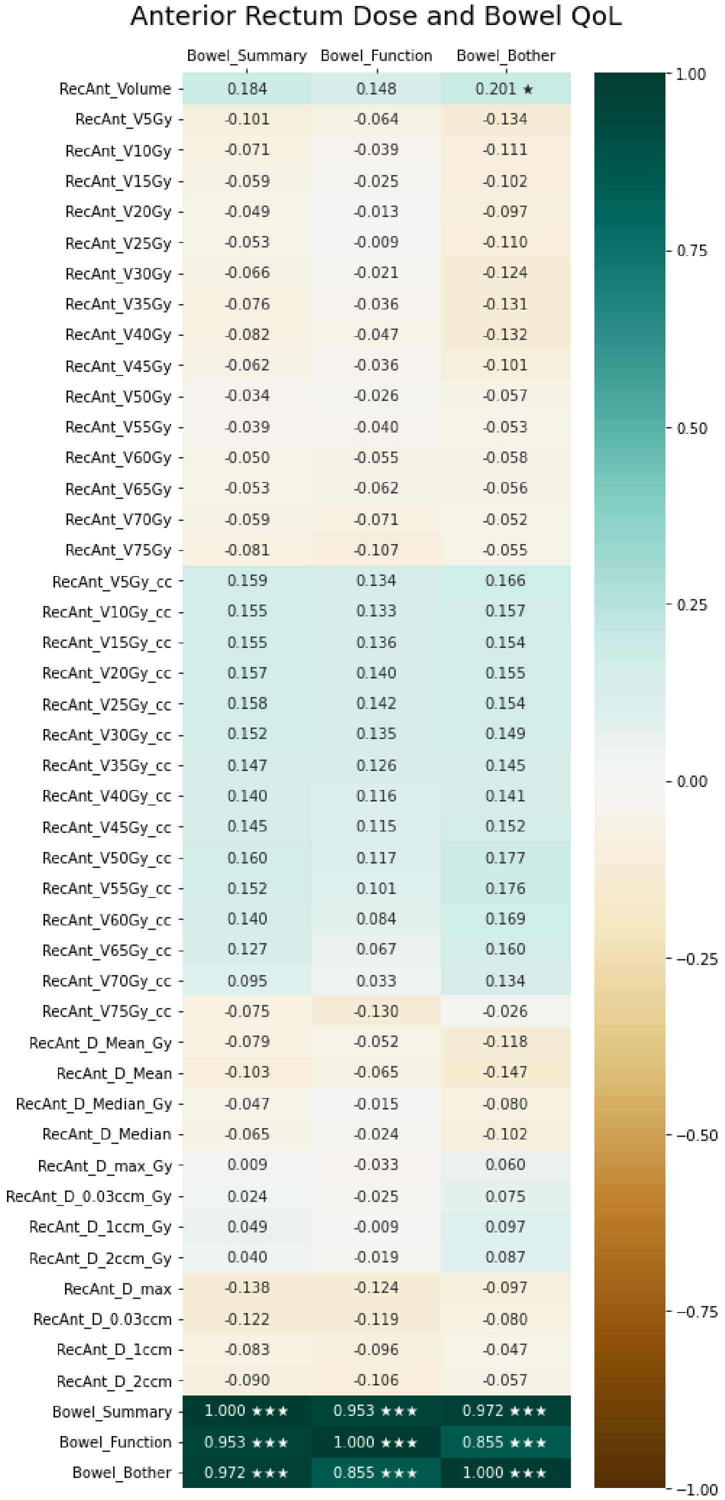

Supplemental Figure S4. Association between anterior rectum dose and bowel-related quality of life.

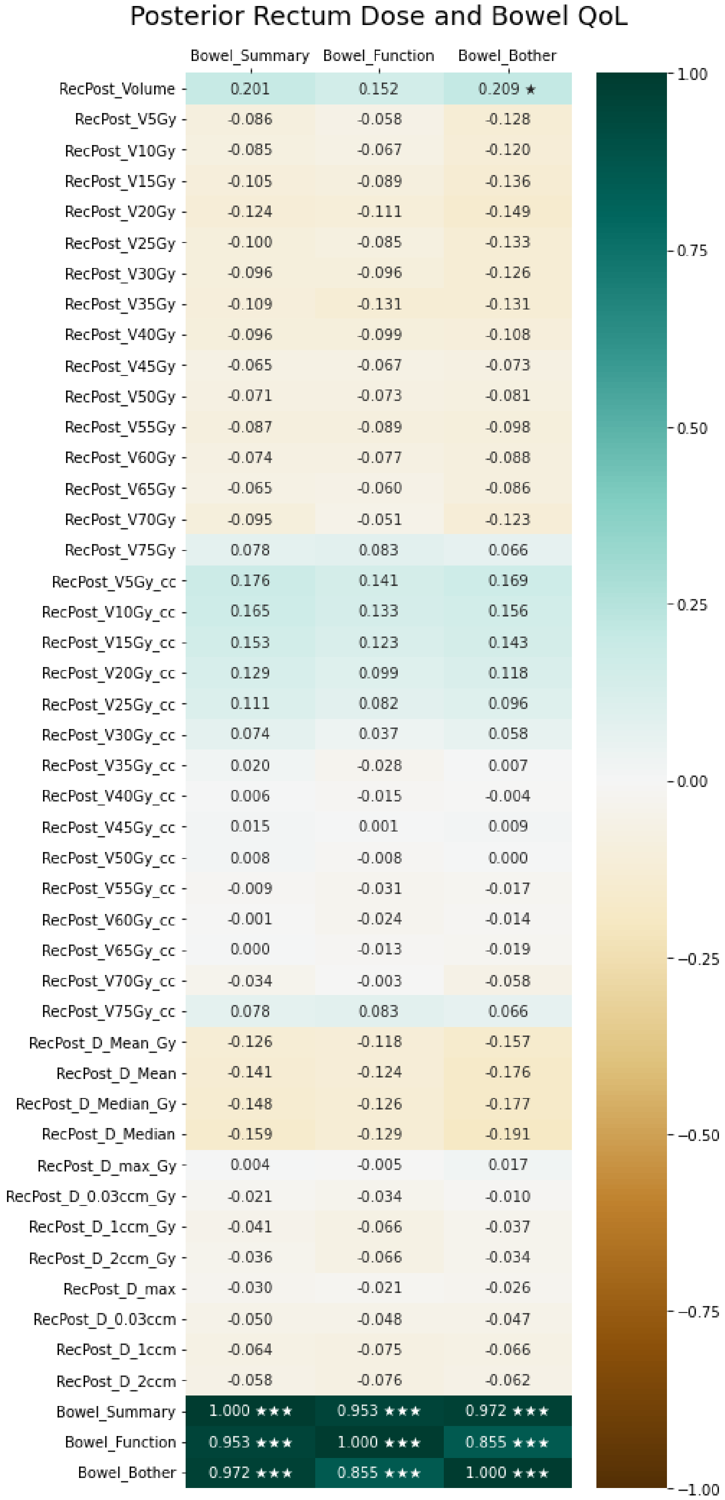

Supplemental Figure S5. Association between posterior rectum dose and bowel-related quality of life.

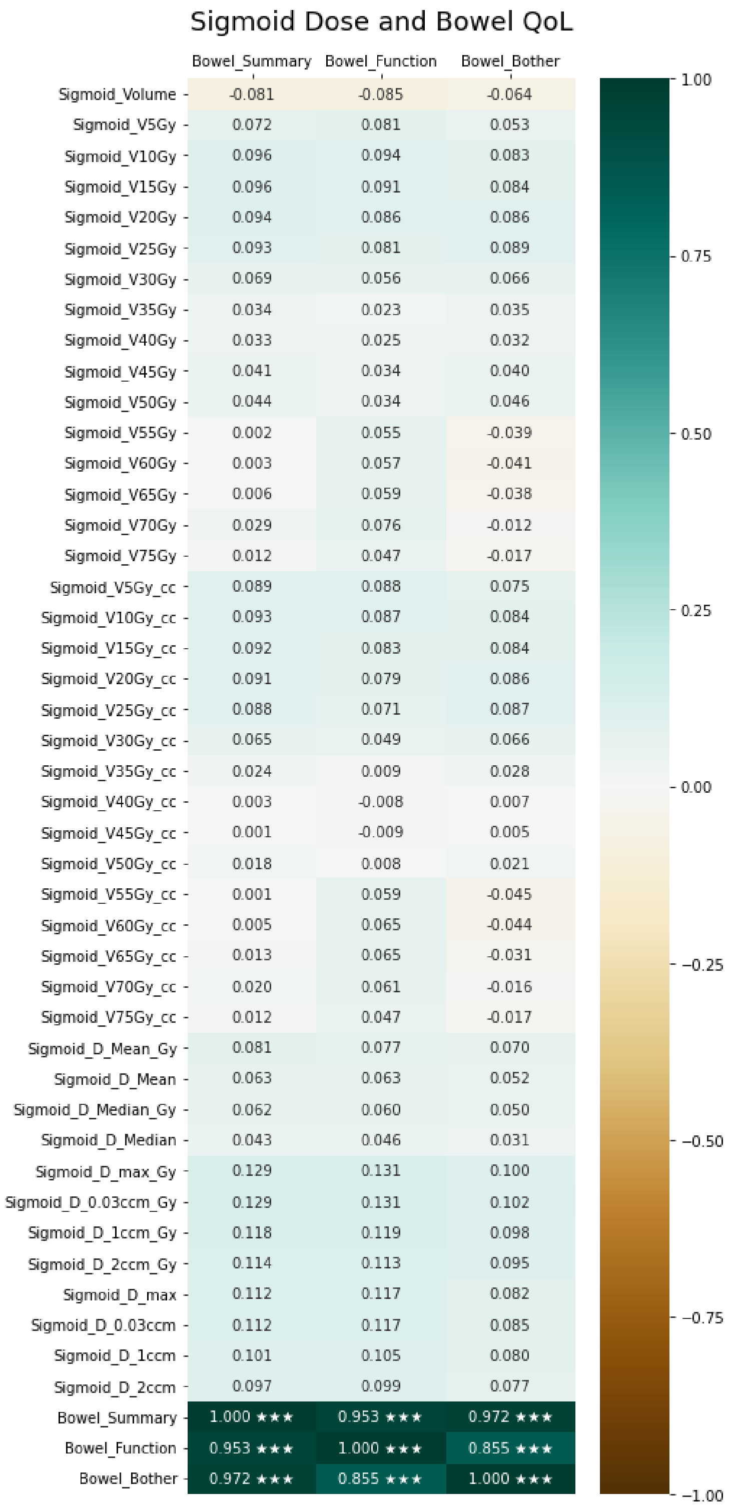

Supplemental Figure S6. Association between sigmoid dose and bowel-related quality of life.
